# Qualitative research on community experiences in large HIV research trials: what have we learned?

**DOI:** 10.1002/jia2.25173

**Published:** 2018-10-17

**Authors:** Carol S Camlin, Janet Seeley

**Affiliations:** ^1^ Department of Obstetrics, Gynecology & Reproductive Sciences Center for AIDS Prevention Studies University of California San Francisco CA USA; ^2^ Department of Global Health and Development London School of Hygiene & Tropical Medicine London UK

**Keywords:** qualitative research, community, randomized controlled trials, publishing, social sciences

## Abstract

**Introduction:**

Very few pragmatic and community‐level effectiveness trials integrate the use of qualitative research over all stages of the trial, to inform trial design, implementation optimization, results interpretation and post‐trial policy recommendations. This is despite the growing demand for mixed methods research from funding agencies and awareness of the vital importance of qualitative and mixed methods research for understanding trial successes and challenges.

**Discussion:**

We offer examples from work we have been involved in to illustrate how qualitative research conducted within trials can reveal vital contextual factors that influence implementation and outcomes, can enable an informed adaptation of trials as they are being conducted and can lead to the formulation of theory regarding the social and behavioural pathways of intervention, while also enabling community engagement in trial design and implementation. These examples are based on published findings from qualitative studies embedded within two ongoing large‐scale studies demonstrating the population‐level impacts of universal HIV testing and treatment strategies in southern and eastern Africa, and a qualitative study conducted alongside a clinical trial testing the adaptation, acceptability and experience of short‐cycle therapy in children and adolescents living with HIV.

**Conclusions:**

We advocate for the integration of qualitative with clinical and survey research methods in pragmatic clinical and community‐level trials and implementation studies, and for increasing visibility of qualitative and mixed methods research in medical journals. Qualitative research from trials ideally should be published along with clinical outcome data, either integrated into the “main” trial papers or published concurrently in the same journal issue. Integration of qualitative research within trials can help not only to understand the *why* behind success or failure of interventions in different contexts, but also inform the adaptation of interventions that can facilitate their success, and lead to new alternative strategies and to policy changes that may be vital for achieving public health goals, including the end of AIDS.

## 1 Introduction

In 2009, Simon Lewin and colleagues published a review on the use of qualitative methods *alongside* research trials of complex healthcare interventions 1. They observed that of the 100 trials reviewed (every fifth trial of the 492 trials listed in the register of the Cochrane Effective Practice and Organisation of Care Review group for 2001 to 2003), 30 had included qualitative work or had research based on qualitative methods associated with them. A number of these trials had made use of qualitative data before and during the trial, but only two had, at that time, integrated the use of qualitative research in all stages of the trial. Reasons given for not using mixed methods included lack of supportive funding and appropriate qualitative expertise as part of the main research team. In recent years, the use of mixed methods in trials has become more accepted by funders 2, 3, 4, 5 and social scientists more likely to be a *part* of the trial team (not working *alongside* the trial) 6, 7, 8.

That said, qualitative methods are often still viewed as contributing to particular aspects of the trial such as informing recruitment of target groups or intervention adherence strategies, measuring and supporting community engagement 9, 10, 11 or contributing to explaining trial results 12, 13, 14, 15. In this commentary, we summarize the key benefits of inclusion of qualitative and mixed methods in trials. We examine the use of qualitative methods for contributing over all stages of the trial, including trial design, optimizing implementation, interpreting results and shaping post‐trial policy recommendations. We advocate for equitable collaborative working across disciplines and in approaches to publication and all forms of dissemination.

We take several examples, based on published sources, from work we have been involved in, to illustrate how integration of qualitative research within randomized controlled trials and implementation studies can reveal vital contextual factors that can influence implementation and outcomes (including both positive and negative unintended consequences), can enable an informed adaptation of trials as they are being conducted and also lead to the formulation of new hypotheses regarding the social and behavioural pathways of intervention action (i.e. theory building). We share examples of qualitative research studies embedded within two large community‐based trials testing the population‐level effects of universal HIV testing and treatment (UTT) (which aims to extend HIV counselling and testing to an entire population and antiretroviral therapy (ART) to all those person living with HIV) in southern and eastern Africa 16, and of a clinical trial of an intervention to test the adaptation, acceptability and experience of short‐cycle therapy 17 in children and adolescents living with HIV.

## 2 Discussion

### 2.1 A UTT intervention trial in Kenya and Uganda

Our first example is taken from the Sustainable East African Research in Community Health (SEARCH) (NCT# 01864603) study, an ongoing community cluster randomized controlled trial (NCT#01864603) in 32 communities of approximately 10,000 persons each located in three regions in Kenya and Uganda. SEARCH aims to evaluate the health, economic and educational impacts of a community‐based strategy for immediate and streamlined ART for all HIV‐positive persons. A longitudinal qualitative research study embedded within the trial aims to reveal social, behavioural and implementation processes that influence the UTT strategy and its outcomes: why the strategy works or fails in communities, and how it operates in diverse settings. The qualitative findings also have been periodically “fed back” to trial leadership and regional teams to explain how the intervention has evolved, and to inform optimization. The SEARCH trial design is adaptive, and newer methods for inference and estimation of “treatment effect” are used 18, which permitted refinements to the intervention design and implementation over time. Thus, inclusion of a longitudinal qualitative study within SEARCH was particularly valuable. Methods include annual in‐depth interviews with cohorts of community members, community leaders and healthcare providers, participant observation at community health campaigns (CHCs) and focus group discussions with CHC attendees. Data collection began in February 2014 and is ongoing.

The SEARCH strategy involved multiple interventions to achieve the UNAIDS 90‐90‐90 targets. To reach the first “90%,” the study conducted multi‐disease testing and services at CHCs combined with home‐based testing for those who did not participate in campaigns 19. The strategy began with community ethnographic mapping (used to define characteristics for pair‐matching) as well as consultative community meetings to ascertain community preferences for certain intervention elements (non‐HIV services, which varied by community, included hypertension and diabetes screening, malaria rapid diagnostic testing, medical male circumcision, cervical cancer screening and other services). To ensure at least 90% of those diagnosed were linked to care, the study used rapid linkage at testing, appointment reminders, improved provider access through telephones and face‐to‐face meetings, and missed appointment tracking. To ensure that 90% of those in care have undetectable viral loads, SEARCH used a “streamlined care” approach designed to lengthen intervals between visits for stable patients, offer shorter waiting times and ensure a friendly environment in clinics 20. SEARCH demonstrated the effectiveness of its model for high HIV “cascade coverage,” and increased population viral suppression from 45% to 81%, exceeding the “90‐90‐90” targets within two years in intervention communities 21. Initially, testing uptake in the study for men was lower than that of women (62% vs. 74%). Early qualitative research findings on the structural and cultural factors that hindered men's participation in testing campaigns 22 helped to explain these observations. The team found that men's livelihoods and mobility meant they were often away from rural homesteads and could not easily access testing campaigns or HIV care during work hours. Gender norms that ran counter to men's care‐seeking, and valorized their risk‐taking, were also said to inhibit their interest in CHCs; many men preferred to “test by proxy,” inferring their own HIV status from their wife's. Qualitative interviews and focus groups revealed that health campaigns and clinics were seen as “female spaces” that men hesitated to enter, despite incentives and other features targeting men. SEARCH responded to these early observations by adapting its approach to mobilizing men for testing. The location and timing of CHCs were adjusted to better meet men's needs, with more campaigns conducted near workplaces and on weekends (including “moonlight CHCs” at Lake Victoria beach landing sites). The resources allocated for home‐based testing (disproportionately preferred by men) at client‐selected locations were increased, while campaigns were redesigned to include more incentives, sports activities and other features targeting men to increase their demand for testing. These included football matches, boat races and live bands at campaigns. Men's “spaces” and services were set up at campaigns, including a “men's tent” offering counselling on male sexuality, urgent care services and linkage to male circumcision. Local formal and informal male community leaders were hired to assist with mobilizing other men.

These efforts yielded positive results vis‐à‐vis community‐wide participation in testing, and also in qualitative findings showed that they precipitated new opportunities and anxieties related to the disclosure of HIV‐positive status among those either newly diagnosed or confronted anew with a need to disclose as a result of the intervention. An analysis of experiences related to disclosure of HIV status in narratives of people living with HIV (PLHIV) from SEARCH published by Maeri and colleagues 23 revealed that HIV‐related stigma in communities during the study's baseline year was perceived to be high by community members. Many individuals resisted disclosure because of anticipated stigma, and there were stark gender inequities in the negative consequences of disclosure, with women more likely than men to experience violence or abandonment by partners as a result of their disclosure of HIV‐positive status. That analysis called for efforts to strengthen capacity in health systems for gender‐sensitive provider‐assisted disclosure to address the differing support needs of men and women.

### 2.2 A trial testing a health system intervention to accelerate ART initiation in Uganda

At the same time, qualitative research in SEARCH using data collected in the first two years of the study provided early signs that norms, beliefs and attitudes related to HIV testing, status disclosure and engaging in HIV treatment were changing. Combining these with data collected from another large randomized controlled trial, the Streamlined ART Initiation Strategy (START‐ART) trial in Uganda (NCT#01810289), an analysis published in this journal by Camlin and colleagues 24 posited an unforeseen pathway of intervention action in strategies that seek to harness the potential of ART to bring about improvements in individual health outcomes for PLHIV and large‐scale reductions in HIV incidence. In that article, authors propose that the advent of widespread testing campaigns and efforts to accelerate antiretroviral “treatment for all” in eastern African communities has precipitated a rapidly expanding shift in how people living with HIV infection view themselves and act in the community to promote better health for other PLHIV. HIV‐related stigma acts to reinforce hierarchies of power and to systemically exclude those less enfranchised from society and render them “invisible.” But narratives from PLHIV in communities and in clinics revealed that whether or not they were remunerated, and whether they encountered other PLHIV in clinics or in communities, PLHIV in Kenya and Uganda have been taking on new roles and self‐conceptualizations that are transforming their “spoiled” or stigmatized identity into a new valorized social identity, finding a moral “redemption” via their public advocacy of HIV testing and treatment. These trials did not foresee or plan for it; but as the benefits of ART embolden more and more PLHIV to openly engage in care, many “advocates for ART” are emerging in communities, actively engaged in encouraging others to test, to enrol in HIV treatment, to adhere to ART regimens and to stay engaged in care. PLHIV are not only creating a renewed, destigmatized subjecthood, but are leading opinions and playing a pivotal role in shaping new social norms and attitudes related to HIV testing and treatment in eastern Africa. These findings have led to a deeper understanding of the community impact of the UTT strategy and presented opportunities to engage and support the unanticipated positive social change.

### 2.3 A UTT intervention trial in Zambia and South Africa

Our next example also illustrates the value of qualitative research for informing trial teams about study communities in the early stages of a trial and for shaping subsequent research. Social science research is integrated into the design of HPTN 017 (Population Effects of Antiretroviral Treatment to Reduce HIV Transmission [PopART]) cluster randomized trial 25 to demonstrate the effects of a UTT strategy, as is the case with SEARCH. In 2013, during the initial selection of the 21 communities in Zambia and South Africa for HPTN 071, rapid qualitative research (termed a Broad Brush Survey 26) was conducted to gather data on each community, prior to the implementation of the trial intervention. While the results of this work are drawn on in a number of publications 27, 28, 29, 30, this example focuses on the work published by Bond and colleagues in 2016 31. For the rapid assessment, a small team of social science researchers spent about two weeks staying in each study community to undertake data collection, using group discussions, key informant interviews and observations. The work was organized in a sequence to ensure the team acquired a good overview of the setting before holding in‐depth interviews and discussions about the “HIV landscape” of the community, including access to HIV prevention and care services. Those data were used to document the social, demographic and economic profile of each site for use by the trial implementation team, and also to conduct analysis of the contextual heterogeneity across sites. The authors analysed the variability in response to HIV interventions early in the trial using first year process indicator data from the trial (2014 to 2015) from four Zambian intervention communities (“Arm A”) along with the qualitative assessment findings 31. The latter data were organized according to four meta‐indicators spanning physical features, social organization, social networks and “community identity” narratives, to facilitate comparison between communities. These indicators were developed by a research group aiming to classify the “capability” of response to change across diverse settings in Rome, Turin, London, Zambia and South Africa 32. Applying the meta‐indicator frame to the HPTN 071 rapid assessment data, Bond and colleagues concluded that combining the two sets of data provided valuable insights regarding which differences between communities were likely to matter for HIV intervention uptake. For example, “social organization” differences that mattered included mobility (primarily for work), young men's work patterns, population variability across different housing types and the presence of HIV stakeholders. These were factors that could be tracked for change over the duration of the trial and be used to help interpret variability in the trial outcomes 29.

### 2.4 A multi‐country trial to develop a treatment intervention for young people living with HIV

The last example is from the BREATHER (PENTA 16) clinical trial. Working across 11 countries (including one centre in Uganda), this trial compared virological control of short‐cycle therapy (five days on: two days off) with continuous EFV‐based ART in 199 children and young people (aged 8 to 24) (70 from Uganda) living with HIV with viral load <50 c/mL to examine adaptation, acceptability and experience of short‐cycle therapy to inform intervention development 17, 33. The social science component was not fully funded within the trial funding, and a parallel grant from a different funder was secured by the social scientists to support the qualitative research in Uganda. The qualitative study consisted of repeat in‐depth interviews with a sample of participants from both arms of the trial, and discussion groups at the end to discuss emerging trial results. The qualitative data showed that while there was a strong preference for the option of short‐cycle therapy, to allow weekends off from treatment, young people from both arms reported frequent medication side effects and occasional missed doses that they had rarely shared with clinical staff 34. The final discussion group allowed participants to voice concerns about the risks of short‐cycle therapy for young people who struggled to adhere to treatment 35. These findings informed the way in which the final trial findings were reported and could provide valuable input for further research. It should be noted that while some of the qualitative study findings were integrated into the “main” trial paper the paper 17 detailing the qualitative findings (which was submitted at the same time as the main trial findings paper) was not accepted for publication. The qualitative findings paper was published later in a different journal 34.

## 3 Conclusions

With increased attention to translating biomedical research advances into clinical practice, policy and population‐level impact (requiring widespread social and behavioural change), there is a demand for incorporation of qualitative methods in pragmatic clinical and community‐level trials and implementation science studies 4. The structure of this methods “mixture” can draw upon existing taxonomies of mixed methods designs 36, 37, but we suggest that the integration of qualitative methods within trials, particularly when applied using constructivist grounded theoretical approaches (e.g. as articulated by Charmaz 38), can allow researchers to not only pursue a set of research questions defined *a priori*, but also generate new avenues of inquiry and opportunities for theory building in response to unexpected empirical findings. Especially in complex trials, interventions are often not implemented as planned, secular trends affect outcomes, and outcomes and their generalizability cannot be interpreted intelligibly without an in‐depth understanding of context. The increasing use of novel adaptive trial designs and hybrid implementation‐effectiveness trial designs is propitious for integration of longitudinal qualitative research, because these designs facilitate use of qualitative findings to inform optimization of interventions as they are being implemented; moreover, these designs value measurement of heterogeneous “implementation” and “contexts,” aiming to elucidate rather than obscure these factors. The integration of qualitative research within trials can help not only to understand the *why* behind success or failure of interventions in different contexts, but also inform the adaptation of interventions that can facilitate their success, and lead to new alternative strategies and to policy changes that may be vital for achieving public health goals.

We advocate specifically for the pairing of qualitative with clinical and survey research methods in trials and implementation studies, and for the publication of qualitative research from trials with clinical outcome data, either fully integrated into the “main” trial papers or published concurrently in the same journal issue. The option of two complementary papers (of equal weight) is probably the most viable, given word limits may preclude adequate coverage of all results in one paper.

The findings from qualitative research within trials offer valuable information on the ways people behave and communicate, and the complex social worlds with which research is conducted – information that is essential to the understanding of trials’ results 6. However, we continue to find that papers based on qualitative methods from trials are afforded lower priority by many medical journals, despite recent efforts to urge editors to reconsider policies towards the publication of such research 39. Social scientists continue to push the boundaries of disciplinary biases in biomedical HIV research and the medical literature, but the advocacy of clinical researchers is essential to achieve widespread awareness that biomedical research is strengthened through the inclusion of social sciences in the centre of its sphere of inquiry.

## Competing interests

The authors have no competing interests to declare.

## Authors’ contributions

The authors contributed equally to the conceptualization, writing and editing of this article.
